# A nationwide telepathology consultation and quality control program in China: implementation and result analysis

**DOI:** 10.1186/1746-1596-9-S1-S2

**Published:** 2014-12-19

**Authors:** Je Chen, Yahui Jiao, Chaohui Lu, Jun Zhou, Zongjiu Zhang, Chen Zhou

**Affiliations:** 1 Pathology Quality Control Center of China, Department of Pathology, Peking Union Medical College, Beijing, China; 2 Division of Quality of Healthcare, Bureau of Health Policy and Regulation, National Health and Family Planning Commission, Beijing, China; 3 British Columbia Cancer Agency, University of British Columbia, Vancouver, Canada

## Abstract

**Background:**

Telepathology may play an important role in pathology consultation and quality control for cancer diagnosis in China, as the country has the largest population of cancer patients worldwide. In 2011, the Pathology Quality Control Center of China and Ministry of Health developed and implemented a nationwide telepathology consultation and quality control program for cancer diagnosis in China. We here report the results of the two-year implementation and experiences.

**Methods:**

the program built an Internet based telepathology platform to connect participating hospitals and expert consultants. The hardware and software used for the platform were validated in previous validation studies in China. The program had three regional centers consisting of Peking Union Medical College, Huasi Medical College of Sichuan and 2nd affiliated hospital of Zhejiang University. It also had 20 provincial consultation centers based in the provincial referral hospitals. 80 provincial or national pathologists served as expert consultants for the program, providing telepathology consultation for cancer diagnosis for more than 60 participating hospitals.

**Results:**

from 2011 to July 2013, 16,247 pathology cases were submitted to the platform for consultation. Among them, 84% were due to diagnostic difficulty and 16% were due to request by patients. The preliminary diagnosis provided by submitting pathologists were in agreement with expert opinion in 59.8% of cases but was in disagreement with expert opinion in 24.2% of cases. 16.0% of cases were not provided with preliminary diagnosis. The distribution of pathology cases by system or organ were: digestive system, 17.3%; gynecologic system, 16.7%; head and neck, 15.7%; bone and soft tissue, 10.4%; lung and mediastinum, 8.6%; breast, 7.6%; urinary system, 7.5%; hematopathology, 6.4%; skin, 5.2%; neuropathology, 2.5% and cytopathology, 1.3%. Expert consultants also provided assessment of quality of slide preparation and staining, online lectures and guidance for pathology quality control.

**Conclusion:**

our results of two years' implementation indicated that telepathology could solve the problem of uneven distribution of pathology resources and provide a solution for countrywide pathology quality control in China. Telepathology could play an important role in improving pathology diagnosis in China.

## Background

WHO estimated that China had 2.80 million newly diagnosed cancer patients in 2008 [1]. According to National Health and Family Planning Commission of China, cancer is the No.1 cause of death in China, accounting for 1.96 million deaths annually. As the most populous country in the world with a population exceeding 1.37 billion, China has the largest number of cancer patients worldwide. In addition, its population is rapidly aging. As the number of newly diagnosed cancer patient in China has been increasing rapidly, cancer diagnosis and treatment are becoming an important part of health care in China.

Accuracy of pathology diagnosis plays a key role in the effective management of cancer patients. China however, has limited pathology resources. The country has limited numbers of well-trained and experienced pathologists; most of them work in large teaching hospitals in big cities. A recent survey by Chinese Pathologists Association showed that a majority of pathologists in clinical practice in China had only one year of formal training. Pathology residency programs similar to those of Western countries are only available since 2010 and are limited in a couple of large medical schools. Most pathologists and even pathologists in large tertiary teaching hospitals or provincial referral hospitals are not sub-specialized. Pathologists in community hospitals often have difficulty in diagnosing rare or complex cancer pathology cases. Error in pathology diagnosis is not uncommon, which often results in medical legal problem. There is an urgent need for pathology consultation and pathology quality control in China.

Telepathology is the practice of pathology by interpreting digitalized images or whole slide images (WSI) by pathologists at a long distance. It could be applied in many areas of pathology practice, including remote consultation, quality control, teaching and continuing medical education [2,3]. Telepathology offers the efficient utilization of subspecialty pathologists to solve problems of shortage of pathologists and subspecialists at remote sites or remote hospitals [4]. In order to improve the quality of pathology diagnosis by using limited pathology resources, the Pathology Quality Control Center of China, under the guidance of National Health and Family Planning Commission, developed and implemented a nationwide telepathology consultation and quality control program for cancer diagnosis in China in 2011. We here report the results of the implementation and two-years operation of the program.

## Methods

### Internet platform and network

A website based telepathology consultation platform http://www.mpathology.cn was developed to provide connections for hospitals and expert consultants. An Internet based platform could be accessed by multiple hospitals/institutions and by expert pathologists from different institutions. The program has three regional centers based in Peking Union Medical College, Beijing, Huasi Medical College of Sichuan, and 2^nd ^affiliated hospital of Zhejiang University respectively, and 20 provincial centers based in their respective provincial referral hospitals. Pathologists from community hospitals sent digital images or WSI of the histology slides of cancer through the internet to the server of the platform to be stored in the server; expert pathologists from different institutions could access the website through the internet, review the WSI stored in the server, make a pathology diagnosis or offer a second opinion, and assess the quality of pathology slides and pathology diagnosis from submitting hospitals.

For telepathology consultation, the following steps are involved: pathologists from community hospitals sent consultation requests accompanied by attached WSI and relevant clinical information to the platform, the system alerts its technician by e-mails; the technician checks the submitted materials for the name of the expert pathologist being requested for consultation and completeness of the submitted images and clinical information; the system manager contacts the expert pathologist by cell phone message and email, informing him or her of pending consultation case. The expert pathologist logs into the website by a computer or an Ipad; checks out the pending cases for consultation under his/her name; review pathology of the WSI; captures a representative image from WSI to included in the final report; and enters the pathology diagnosis. Once the expert pathologist releases the final report, the system automatically informs its technician who then sends pathology consultation report to submitting pathologist by email and regular mail.

For quality control, expert pathologists logged into the website to assess the accuracy of pathology diagnosis and quality of histology slides of specific hospitals. The results of the assessment were sent to provincial and national pathology quality control centers.

All participating hospitals were equipped with a virtual microscope (Motic Medical Diagnostic System, China). The microscope and its software were designed to scan histology glass slides, generate and integrate digital images into a whole slide image (WSI). A WSI of an H/E tissue slide under x20 objective (0.47 µm/pixel) could be completed within 3 minutes. The WSI could be viewed as if it is a tissue slide under a microscope with 2×, 4×, 10×, 20× and 40× objectives. The virtual microscope and related software were tested and validated in a previous study of a variety of 600 surgical pathology specimens in China. The study showed a high concordance rate (94.2%) between WSI and H/E glass slide [5,6].

### Expert consultants and hospitals

All of the 80 expert pathologists were selected from 356 pathologists, who had provincial and national reputations and volunteered to participate in the project. The pathologists took an online assessment of 30 pathology consultation cases in WSI format. Based on the assessment result, 80 pathologists were selected as expert consultants for the program. The names, location and expertise of the pathologists were listed on the website http://www.mpathology.cn.

60 out of 87 hospitals in 20 provinces were selected for the program. Pathologists from the hospitals took an online diagnostic test consisting of 50 pathology cases. Those hospitals having pathologists with poor test results were included in the program. All of the participating hospitals have more than 6000 surgical pathology cases annually.

### Training and quality controls

In September 2011, expert pathologists in three regional centers held a short-term training session for pathologists and laboratory technologists from 57 participating hospitals. The training included tissue grossing and processing, preparation of H/E histology slide, digital scanning of histologic slide, and application of telepathology software to create WSI, manage case files, connect to the website of the platform. Privacy policy and secure storage of the WSI and teleconsultation reports were emphasized. In March, August and October 2012, expert pathologists inspected 22 participating hospitals in three separate visits, providing onsite assessment and guidance to improve the accuracy and quality of pathology diagnosis of the hospitals.

## Results

**Figure 1:**
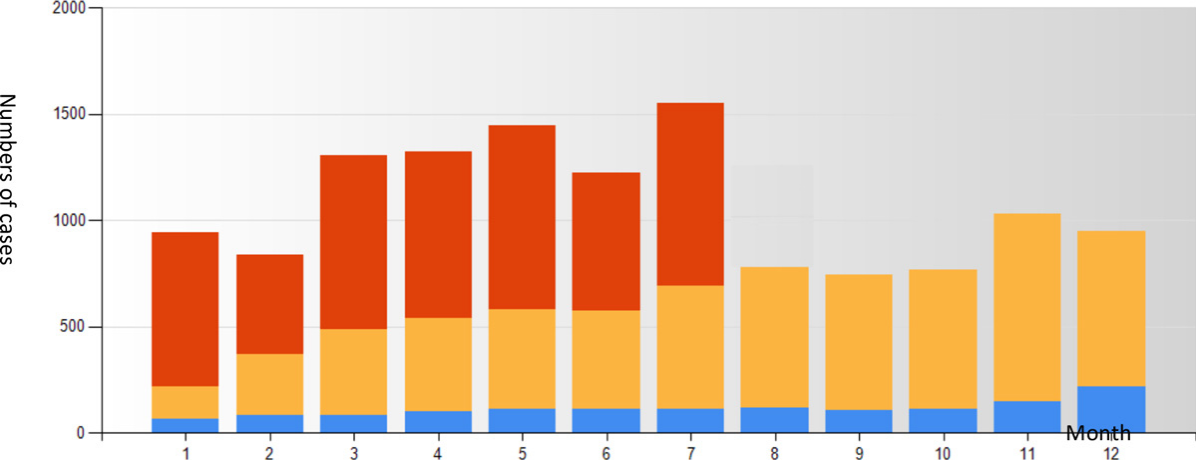
**Numbers of consultation cases in different years**.

Figure 1 shows that the number of pathology consultation cases sent to the program increased significantly from 2011 to 2013. From the inception of the program to July 2013, a total of 16,247 pathology cases were sent to the platform for consultation. Among them, 84% were cases with diagnostic difficulty; 16% were consultation requested by the patients. Hospitals located in urban areas sent in 12,510 cases, accounting for 77% of cases. Hospitals located in county or rural areas sent in 3,737 cases, accounting for 23% of cases.

Table 1 shows the turn-around time for pathology consultation in the telepathology platform. 87% cases had consultation reports released within 48 hours, and 61.5% cases had consultation reports released within 24 hours.

**Table 1:** Turn around time of the teleconsultation.

*Time (hours)*	*Case Number*	*Percent (%)*
<24	9,992	61.5
24-48	4,143	25.5
>48	2,112	13.0

**Figure 2:**
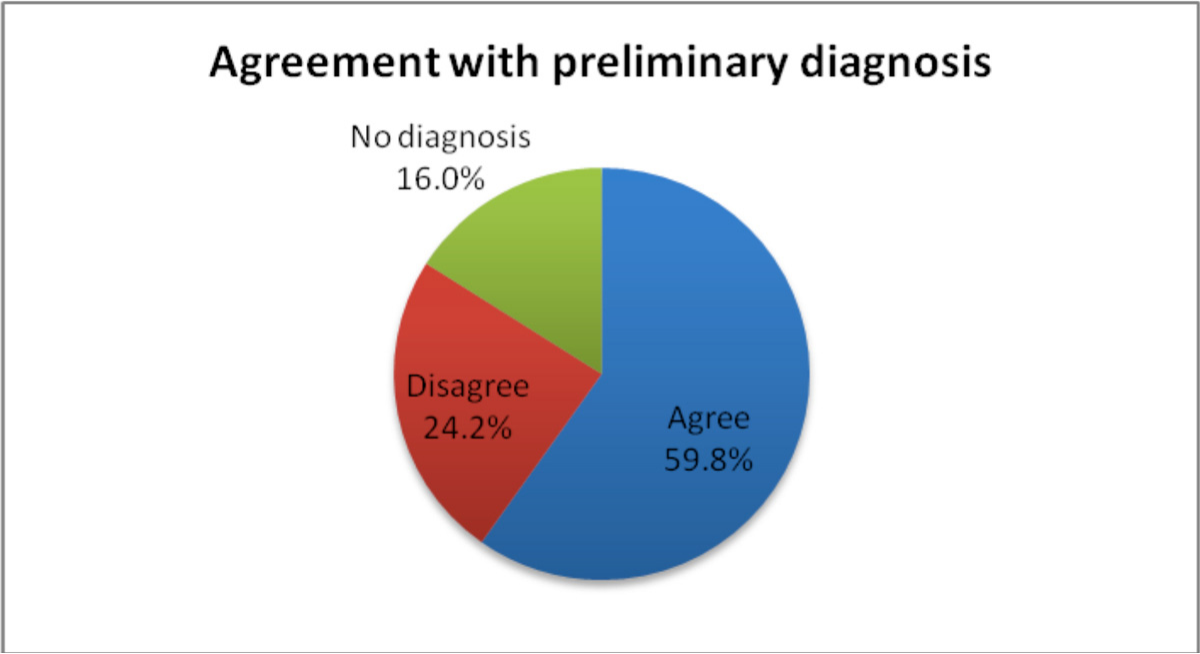
**Agreement of pathologic diagnosis between expert consultants and submitting pathologists**.

Figure 2 shows the agreement of diagnosis between expert consultants and submitting community pathologists. Expert consultants agreed with preliminary diagnosis in 59.8% of cases but disagreed with preliminary diagnosis in 24.2% of cases. Submitting pathologists did not provide preliminary diagnosis for 16% of cases.

Table 2 shows the distribution of consultation cases among different body system and organs: digestive system, 17.3%; gynecologic system, 16.7%; head and neck, 15.7%; bone and soft tissue, 10.4%; lung and mediastinum, 8.6%; breast, 7.6%; urinary system, 7.5%; hematopathology, 6.4%; skin, 5.2%; neuropathology, 2.5% and cytopathology, 1.3%.

**Table 2:** Distribution of consultation cases among different body organs and systems.

*Body system*	*Percent (%)*
Digestive, liver and pancreas	17.3
Gynecologic	16.7
Head and neck	15.7
Bone and soft tissue	10.4
Lung and mediastinum	8.6
Breast	7.6
Urinary system	7.5
Hematopathology	6.4
Skin	5.2
Neuropathology	2.5
Cytology	1.3
Neuroendocrine	0.5
Cardiovascular	0.1
Total	100.0

## Discussion

In recent years, telepathology has been applied successfully in remote pathology consultation and in quality control [2,7]. Since 2009, because of the availability of high speed Internet, 3G wireless networks and low cost "made in China" commercial virtual microscopes, telepathology has become the focus of attention among pathology community in China. A validation study of telepathology consultation in China showed that the mean accuracy of pathology diagnosis using virtual image is 94.2% as compared to H/E glass slide [5]. In 2010, Ministry of Health of China released an announcement to promote telepathology for cancer diagnosis in China and initiated a plan to build a nationwide telepathology consultation and quality control network. The goal is to provide telepathology consultation and to monitor the accuracy of pathology diagnosis of cancer in China. The program called for provinces to develop their own provincial telepathology consultation and quality control centers, each of them then connected to national center. By 2011, a website based consultation and quality control platform were implemented to connect a network of 60 hospitals from 20 provinces.

The results of the two-year experience and implementation showed that the number of consultation cases submitted to the platform increased quickly. Within one and half year, a total number of 16,247 cases have been reached. Most of the cases were diagnostic challenging cases. More cases were sent by hospitals from urban areas as compared with hospital from county/rural areas. The reason may be due to that hospitals in urban areas have more surgical pathology specimens and more pathology cases with diagnostic complexity as compared to county/rural areas. Another reason is that the pathologists and patients in urban hospitals were better informed about telepathology consultation platform than those in county/rural hospitals. Our results indicated that telepathology could play a role in cancer pathology diagnosis in both county/rural hospitals and city hospitals.

Analysis of the turn-around time of telepathology consultation showed that almost all of our consultation reports were released within 48 hours. Our turn around time is much shorter than the 7 average days of turn around time of conventional pathology consultation reported in US where glass slides were used [8]. A shorter turn-around time for cancer diagnosis reduces the anxiety of patients and increases the chance for immediate treatment. In view of turn-around time, consultation provided by telepathology has great advantage over conventional pathology consultation.

Pathology consultation or second opinion in pathology diagnosis plays a pivotal role in cancer diagnosis [9,10]. Several reports from North America showed that the disagreement in pathology diagnosis between expert consultants and submitting pathologists were around 10% [11,12]. The disagreement in diagnosis for lymphoma was as high as 16.4% [13]. Among Asian countries, the rate of disagreement in pathology diagnosis between expert pathologists and submitting pathologists is much higher, ranging from 16% to 64.3% [14,15]. An analysis of 673 consultation cases sent to Twain Cancer Center showed that 16% of the cases had second opinion significantly differ from primary diagnosis [14]. Hsu et al reported that, among 2,686 consultation cases, 64.3% of cases had expert opinion different from preliminary diagnosis and 205 cases (12.3%) showed significant disagreement [15]. Our result of 16,247 teleconsultation cases showed that 24.2% (3,932 cases) of cases had experts' opinion significantly different from submitting pathologists. The significant difference means a change from malignant to benign or from benign to malignant. The rate increases to 28.8% (3,932 out of 13,647 cases) if cases without preliminary diagnosis were excluded from analysis. Our result is significantly high than those reported in North America and Twain but lower than those reported in other Asia countries or region. In addition, 16% of our cases were received without a preliminary diagnosis provided by community pathologists, which indicated that 40.2% of our cases benefit from telepathology consultation. Our results indicated that telepathology is very valuable in cancer diagnosis in China.

Due to the differences in cancer types and in biopsy rate, the distribution of consultation cases among different body organs and systems may be different between Western countries and China. Our results showed that in China, most of the consultation cases were from gastrointestinal tract/live and pancreas, gynecology, head and neck, bone and soft tissue, and respiratory system. Unlike Western countries, skin pathology, hematopathology and cytopathology accounted for low percentage of the consultation cases in China [10]. The difference in distribution of teleconsultation cases may provide guidance for training of subspecialty pathologists in China.

From May 2012, the program provided more than 40 Internet based pathology lectures by expert consultants. The lectures are free and open to all pathologists in the country. The result indicated that the platform could also play a role in providing continuing medical education for pathologists nationwide.

In future, telepathology or digital pathology will play a key role in pathology diagnosis and quality control, as its current development is accelerate by the advance of digital and internet technology, and is supported by the government. Telepathology is also cost effective and could play a role in health care reform. Telepathology consultation aids pathologists to diagnose difficult pathology cases, reduces errors in diagnosis and avoids unnecessary medical legal expenses; telepathology also benefits patients who no longer need to travel to large hospitals for a second opinion for cancer diagnosis, thus saving time and money for patients. We believe that in near future telepathology will become an important part of telemedicine and will play a role in health care reform in China.

## Conclusion

We reported the implementation of a nationwide telepathology consultation platform in China and analyze the results of its two-year operation. An internet based telepathology open platform was established to connect 60 participating hospitals and 80 pathology experts. Our two-years results indicated that 16, 247 pathology cases were sent to the platform over one and half years. Telepathology provided a fast consultation service with a turn around time less than 48 hours and improved cancer pathology diagnosis in about 40% of cases submitted for consultation. Telepathology could play an important role in improving quality of cancer diagnosis and in solving a shortage of pathology resource in China.

## Competing interests

The authors declare that they have no competing interests.

## Authors' contributions

Dr. Je Chen designed and chaired the telepathology platform. He also guided the analysis and writing of the manuscript. Dr. Yahui Jiao participated in maintenance of the telepathology platform, analysis of the results and writing of the manuscript. Dr. Chaohui Lu participated in the design, implementation and maintenance of the telepathology platform. He also participated in analysis of the results. Dr. June Zhou participated in implementation and maintenance of the telepathology platform. Dr. Zongjiu Zhang designed and guided implementation and maintenance of the telepathology platform. Dr. Chen Zhou analyzed the results of telepathology consultation and wrote the manuscript.

## Notes

Publication of this supplement has been funded by 12th European Congress on Digital Pathology. This article has been published as part of *Diagnostic Pathology *Volume 9 Supplement 1, 2014: Selected articles from the 12th European Congress on Digital Pathology. The full contents of the supplement are available online at http://www.diagnosticpathology.org/supplements/9/S1
